# All-a-glow: spectral characteristics confirm widespread fluorescence for mammals

**DOI:** 10.1098/rsos.230325

**Published:** 2023-10-04

**Authors:** Kenny J. Travouillon, Christine Cooper, Jemmy T. Bouzin, Linette S. Umbrello, Simon W. Lewis

**Affiliations:** ^1^ School of Molecular and Life Sciences, Curtin University, Bentley, Western Australia 6102, Australia; ^2^ Collections and Research, Western Australian Museum, Welshpool, Western Australia 6106, Australia; ^3^ School of Biology and Environmental Science, Queensland University of Technology, Gardens Point Campus, 2 George Street, Brisbane, Queensland 4001, Australia

**Keywords:** UV light, fluorescence, mammal, fur, skin

## Abstract

Mammalian fluorescence has been reported from numerous species of monotreme, marsupial and placental mammal. However, it is unknown how widespread this phenomenon is among mammals, it is unclear for many species if these observations of ‘glowing’ are true fluorescence and the biological function of fluorescence remains undetermined. We examined a wide range of mammal species held in a museum collection for the presence of apparent fluorescence using UV light, and then analysed a subset of preserved and non-preserved specimens by fluorescent spectroscopy at three different excitation wavelengths to assess whether the observations were fluorescence or optical scatter, and the impact of specimen preservation. We also evaluated if fluorescence was related to biological traits. We found that fluorescence is widespread in mammalian taxa; we identified examples of the phenomena among 125 species representing all 27 living mammalian orders and 79 families. For a number of model species, there was no evidence of a corresponding shift in the emission spectra when the wavelength of excitation was shifted, suggesting that observations of ‘glowing’ mammals were indeed fluorescence. Preservation method impacted the intensity of fluorescence. Fluorescence was most common and most intense among nocturnal species and those with terrestrial, arboreal and fossorial habits, with more of their body being more fluorescent. It remains unclear if fluorescence has any specific biological role for mammals. It appears to be a ubiquitous property of unpigmented fur and skin but may function to make these areas appear brighter and therefore enhance visual signalling, especially for nocturnal species.

## 1. Introduction

Fluorescence is the process by which a chemical (e.g. protein, carotenoid) on the surface of an organism absorbs light and then emits the light at longer and lower-energy wavelengths [1]. One example is when an animal's surface absorbs high-energy, short-wavelength ultraviolet (UV) light and emits the fluorescence as a lower-energy coloured, often pink, green or blue, glow. Fluorescence can make the previously invisible UV light visible by shifting it within the range of white light, so an animal does not necessarily have to see into the UV spectrum to detect fluorescence [2]. Numerous organisms have been reported to fluoresce including plants [3], corals [4], insects [5], spiders [6], scorpions [7], crustaceans [8], molluscs [9], fish [10], amphibians [11], reptiles [12] and birds [13]. Fluorescent compounds have been identified in a variety of animal materials including bone, teeth, claws, fur, feathers, carapace and skin, and the visible fluorescent colours observed include red, yellow, green, blue and pink [2,12–18]. The reported evolutionary functions for this fluorescence are varied and include the enhanced camouflage [19], signalling to conspecifics including mate signalling [9,13], threat displays to predators and conspecifics [8], enhanced photosynthesis [4] and environmental marking [20].

Among mammals, the first published reports of fluorescence were in leporids (e.g. rabbits) and hominids (humans) by Stübel [21] with more recent published observations of fluorescence for New World flying squirrels [22], springhares [23], platypus [24], dormice [25] and a variety of other rodents [26], as well as an array of other mammalian species [2,18,27]. There is also a plethora of anecdotal reports of mammals glowing under UV light [e.g. 28–31]. The occurrence of fluorescence across the three major subdivisions of mammal (monotreme, marsupial and placental mammals) suggests it may be an ancestral trait [24]. To date, the majority of mammals with reported fluorescence are nocturnal, and fluorescence was not observed among some diurnal sciurids [2,22], leading to the hypothesis that fluorescence might be useful at night [22–24] or that fluorescent pigments of diurnal species might photodegrade [18]. There may be other functions for this trait yet to be explored for mammals, as has been proposed for other organisms, or fluorescence may have no specific biological function, being simply a consequence of pigment or other surface structural characteristics [1,26]. The reddish UV photoluminescence (= fluorescence) found in nocturnal mammals is thought to be caused by the presence of free-base porphyrins, which are photodegradable and are unlikely to have a specific function, but rather a by-product of physiological processes [18]. To address these hypotheses regarding the evolutionary history and potential biological role of mammalian fluorescence it is necessary to examine the presence of fluorescence over a large number of mammalian species representing the major families and a variety of ecological niches.

Fluorescence spectroscopy is one of the main techniques used to study fluorescence in mammals. For example, fluorescence spectra of apparently fluorescent body regions of some mammals, including springhare and platypus [18,23,24] have been reported. One factor that needs to be considered when interpreting fluorescence spectra is the phenomenon of light scattering, which can produce similar spectra [32]. Light scattering generally involves the interaction of the irradiated incident light with particles (molecules, atoms) of the sample, deflecting the incident light, and in some cases, the scattered light is emitted at a longer and lower energy wavelength [32]. Because both fluorescence and light scattering involve irradiation of the sample and emission of light at a lower energy, this can lead to interference between the two phenomena. Consequently, when carrying out fluorescent spectroscopy measurements, it is important to establish whether the signal observed is due to fluorescence or light scattering. Apart from Toussaint *et al*. [18] there are few/no studies confirming the origin of the apparent ‘glow’ in mammals is from fluorescence and not from scattering. Another potential source of the apparent fluorescence are the chemicals used to preserve museum specimens. The impact of preservation chemicals was discounted by some as fluorescence was also observed in living specimens [22,23] and for dormice was more intense for living than for dead or preserved specimens [25], but it remains unclear if different museum preservation techniques impact apparent fluorescence for other species.

Here we examine the phenomenon of mammalian fluorescence to determine the extent of apparent fluorescence among mammals and to identify the anatomical regions most likely to fluoresce. We evaluate if the observed ‘glow’ from several apparently fluorescent mammals is actual fluorescence as opposed to optical scatter and assess the likelihood of past (use of arsenic) and present (use of borax) museum specimen preservation techniques contributing to these observations. We interpret our results in an evolutionary and ecological context and consider possible roles of fluorescence among mammals.

## 2. Material and methods

Both preserved and frozen mammal specimens were obtained from the Western Australian Museum collection, with additional freshly frozen specimens of platypus (*Ornithorhynchus anatinus*) and Tasmanian devil (*Sarcophilus harrisii*) obtained from the Tasmanian Museum and Art Gallery, and koala (*Phascolarctos cinereus*) and echidna (*Tachyglossus aculeatus*) from Yanchep in Perth, Western Australia. Phase 1 of the study, the forensic light source and fluorescence spectroscopy, was conducted at Curtin University, while phase 2, the wide-ranging survey of fluorescence, was conducted at the Western Australian Museum. Details of specific species and sample sizes involved in each phase are as indicated below. Note that the museum has kept no records of fumigation history, arsenic soap was used in old specimens pre-1980s, and borax was used in more recent specimens post-1980s.

### 2.1. Phase 1: preliminary considerations, forensic light source studies and fluorescence spectroscopy

The first phase of our study aimed to (a) identify if we could reproduce the findings of previous studies e.g. Anich *et al*. [24] and Toussaint *et al*. [18], (b) determine if apparent fluorescence is truly fluorescence and not light scattering, (c) identify the anatomical regions most likely to fluoresce, and (d) identify if preservation method impacts on fluorescence.

#### 2.1.1. Material for preliminary considerations

The specimens used for phase 1 consisted of two monotreme species, the platypus (one specimen preserved with arsenic powder and a frozen specimen which was analysed, preserved with borax and then re-analysed) and short-beaked echidna (one preserved and one frozen), five marsupials, the koala (one preserved and one frozen), Tasmanian devil (one preserved and one frozen), greater bilby (*Macrotis lagotis*; one preserved and one frozen), quenda (*Isoodon fusciventer*; one preserved) and southern hairy-nosed wombat (*Lasiorhinus latifrons*; one preserved) and a placental mammal, a cat (*Felis catus*; one frozen). Preserved specimens had generally been prepared with arsenic soap and powder (on the inside of the skin), but we included platypus specimens prepared with arsenic and with borax (sodium borate), and the quenda was preserved only with borax, allowing for comparison between the main dry preservation methods. Frozen specimens (freshly dead animals that were not yet preserved with chemicals) were thawed to room temperature and the fur dried with a hair dryer before measurement. Note that preservatives were only used on the inside of the specimens, not on the outside and no insecticides have been used on any specimen.

#### 2.1.2. Forensic light source studies

**Figure 1.**
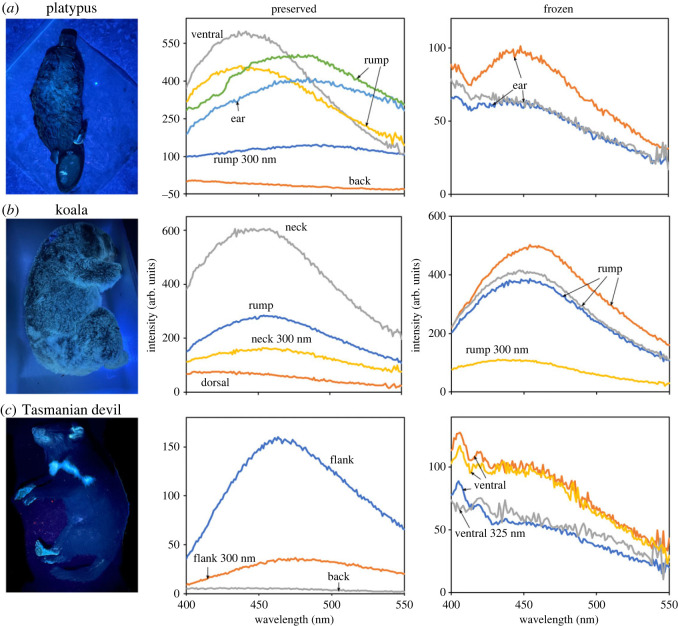
Fluorescence spectra for preserved and frozen specimens of platypus (a), koala (b) and Tasmanian devil (c) at 350 nm exposure, unless stated otherwise. Photo shows frozen specimens under UV light, using a filter.

**Figure 2.**
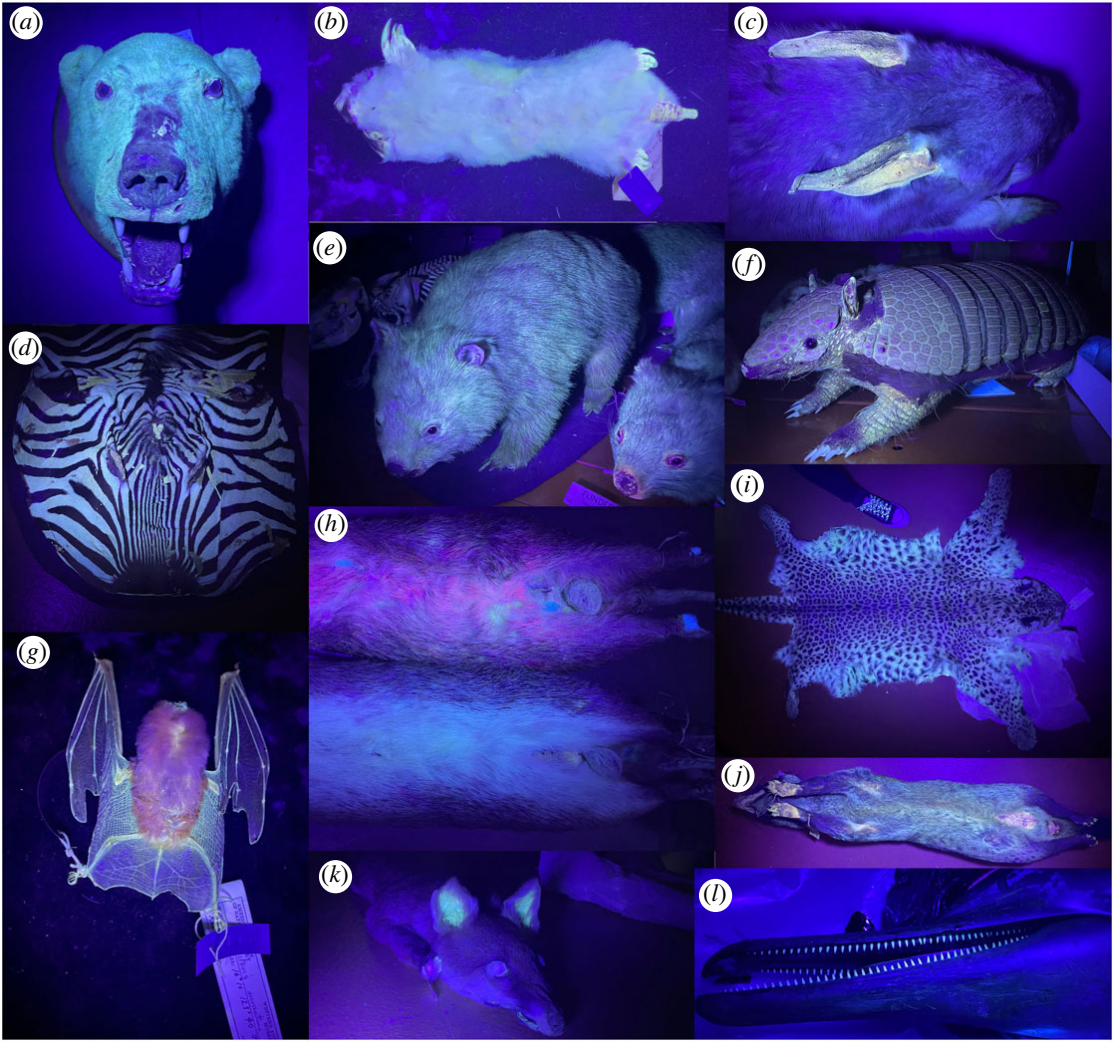
Unfiltered photography of mammal taxidermy under UV light. (a) polar bear, (b) southern marsupial mole, (c) greater bilby, (d) mountain zebra, (e) bare-nosed wombat, (f) six-banded armadillo, (g) orange leaf-nosed bat, (h) quenda, (i) leopard, (j) Asian palm civet, (k) red fox, (l) dwarf spinner dolphin.

The specimens involved in phase 1 were examined using a forensic light source (Rofin Polilight PL500) at 350 nm excitation wavelength and photographed using a Nikon D300 camera, fitted with a 60 mm lens, on manual exposure mode (settings of ISO-200 and a range of f-stops from f11 to f20 and shutter speeds of 3–5 s, due to the difference in specimen size). Photographs were taken with (figure 1) and without (figure 2) with longpass viewing filters (Schott glass 515, 550 and 613 nm placed in front of the camera); the best result was obtained without the filter. The photographs were used to identify likely fluorescent and non-fluorescent regions for subsequent fluorescence spectroscopy analysis (see figure 1).

#### 2.1.3. Fluorescence spectroscopy

Fluorescence spectroscopy experiments were performed using a Cary Eclipse Fluorescence Spectrophotometer (Agilent) combined with a fibre optic probe for *in situ* measurements. For each specimen, spectra were collected from body regions that appeared to fluoresce under UV light and at least one non-fluorescing area, including the dorsal, lateral and ventral fur/spines, any light-coloured areas (e.g. white patches of the Tasmanian devil and koala) and areas of exposed skin on the face, ears or feet. A small (approx. 5 mm^2^) area of each region was excited at 350 nm, and the mean of 10 replicates of the fluorescence spectra collected between 400 and 550 nm with a slit width of 5 nm was recorded. To examine whether the glowing regions were fluorescence or light scatter, spectra were also collected at 300 and 325 nm excitation wavelength for each region. For fluorescence, no shift is expected in the fluorescence spectrum when the excitation wavelength is varied, since the emission wavelength is independent of the excitation wavelength. However, for light scattering, a corresponding shift is expected in the emission peak if the excitation wavelength is changed, since the wavelength of the scattered photons is proportional to the excitation wavelength [32]. A borax sample was also examined under similar conditions.

### 2.2. Phase 2: wide-range survey of fluorescence from specimens in a museum collection

The second phase of our study aimed to (a) identify how widespread fluorescence is in mammals and (b) identify if there are correlations between (i) which part of the animals fluoresce and (ii) their ecology as there have been suggestions that fluorescence may be related to ecological traits such as microhabitat use and activity patterns [18,26].

#### 2.2.1. Material for wide-range survey of fluorescence

We included both dry preserved and frozen unprepared specimens from the Western Australian Museum collection. A total of 146 specimens from 125 species of mammal were examined, representing 79 families and 27 orders from all three mammalian subdivisions (monotremes, marsupials and placentals; specimens examined are listed in electronic supplementary material, tables S1 and S2). Kohler *et al*. [22] found little intraspecific variation between 109 individuals of flying squirrel specimens, so we did not expect a level of intraspecific variation great enough to affect the results of the species we studied. In most cases we did shine the UV lights on many specimens of the same species to check this, and since we saw no difference between specimens, we recorded only a few specimens as exemplars of their species for this study. Sexual dimorphism in fur fluorescence also seems to be uncommon. Therefore, we do not expect other members of the species to differ significantly from the representative specimen we examined.

#### 2.2.2. Photography and observation

All specimens in phase 2 were photographed (see figure 2) under 390 nm UV light from a Crime Scene Tools light source (Xenopus Electronix; XeLED-Cr7UV-390-K). For each specimen, we recorded the occurrence of fluorescence, which part of the specimen fluoresced and the colour (electronic supplementary material, table S1). The anatomical area of the apparent fluorescence on each specimen was recorded in a presence (1) or absence (0) matrix (electronic supplementary material, table S2). The anatomical regions and features examined were facial fur, dorsal fur, dorsal fur pattern (spots or stripes) if present, lateral fur, ventral fur, tail fur, claws, inside of ears, underside of feet and other exposed skin (naked tail, naked skin, pouch, etc.).

#### 2.2.3. Analysis of the fluorescence presence

Ecological traits, including activity period (diurnal, *n* = 71 or nocturnal, *n* = 54), locomotion/habitat (terrestrial, *n* = 62; arboreal, *n* = 42; flying, *n* = 10; burrowing, *n* = 4; aquatic, *n* = 7) and diet (carnivore, *n* = 46; omnivore, *n* = 26; herbivore, *n* = 53), were identified for each specimen after Van Dyck & Strahan [33] and Wilson & Reeder [34]. Potential differences in the frequency of occurrence of the fluorescence for various body parts and of various florescent colours was assessed with a uniform goodness of fit test conducted with statisti*XL* v. 2.2 (www.statistiXL.com, Nedlands, WA). A principal coordinates analysis (PCO), with Dice similarity index, was performed on the fluorescence matrix (electronic supplementary material, table S2) for each of the biological traits using the software PAST (v. 4.10; [35]), and then a linear discriminant analysis (achieved using statisti*XL*) was conducted with the resulting PCO values to test the statistical significance of separation of fluorescence characteristics of species with varying ecological traits.

## 3. Results

### 3.1. Phase 1: preliminary considerations, forensic light source studies and fluorescence spectroscopy

For the platypus, koala and Tasmanian devil the areas with the highest degree of fluorescence were the pale and white fur on the ventral surface (platypus and koala) or neck and rump (Tasmanian devil) with little to no fluorescence for areas with dark fur (figure 1, electronic supplementary material, figures S1–S3). When excited at 350 nm, the spectra of the fluorescing areas peaked at around 450 nm, which correlated with the blue fluorescence observed in the photographs illuminated by the forensic light source. When excited at 325 or 300 nm, the peak remained at 450 nm but the intensity dropped. The overall intensity of the fluorescence was also higher for the koala and Tasmanian devil compared with the platypus. There was no difference in the wavelength of the spectra peaks for frozen compared with preserved specimens for the koala although the intensity was higher for preserved specimens. For the platypus, both preservation methods (arsenic versus borax) produced similar results of higher intensity fluorescence compared with the frozen specimen (electronic supplementary material, figure S3). The white quills and the skin of the pouch of the short-beaked echidna were fluorescent (electronic supplementary figure S4). The pale fur of the southern hairy-nosed wombat (dorsal side) and quenda (ventral side) had the highest intensity fluorescence detected (over 700 arb. units) of the preserved specimens examined (electronic supplementary material, figure S5). The preserved greater bilby specimen (electronic supplementary figure S6) had similar low-intensity fluorescence to the platypus for its skin and white fur, but when exposed to 600 nm, it had much higher fluorescence intensity (over 400 arb. units). This differed for the frozen bilby specimen with much higher fluorescence intensity (up to 600 arb. units) than the preserved bilby. The dark fur on the cat was not fluorescent, but the white fur was, with similar intensity (approx. 200 arb. units) to the platypus (electronic supplementary material, figure S7). Borax powder was fluorescent with a maximum intensity above 400 arb. units, but the intensity dropped when subjected to 325 nm, with two high-intensity peaks at 440 and 540 nm (electronic supplementary material, figure S8).

### 3.2. Phase 2: wide-range survey of fluorescence from specimens in the Western Australian Museum collection

#### 3.2.1. Photography and observation

**Figure 3.**
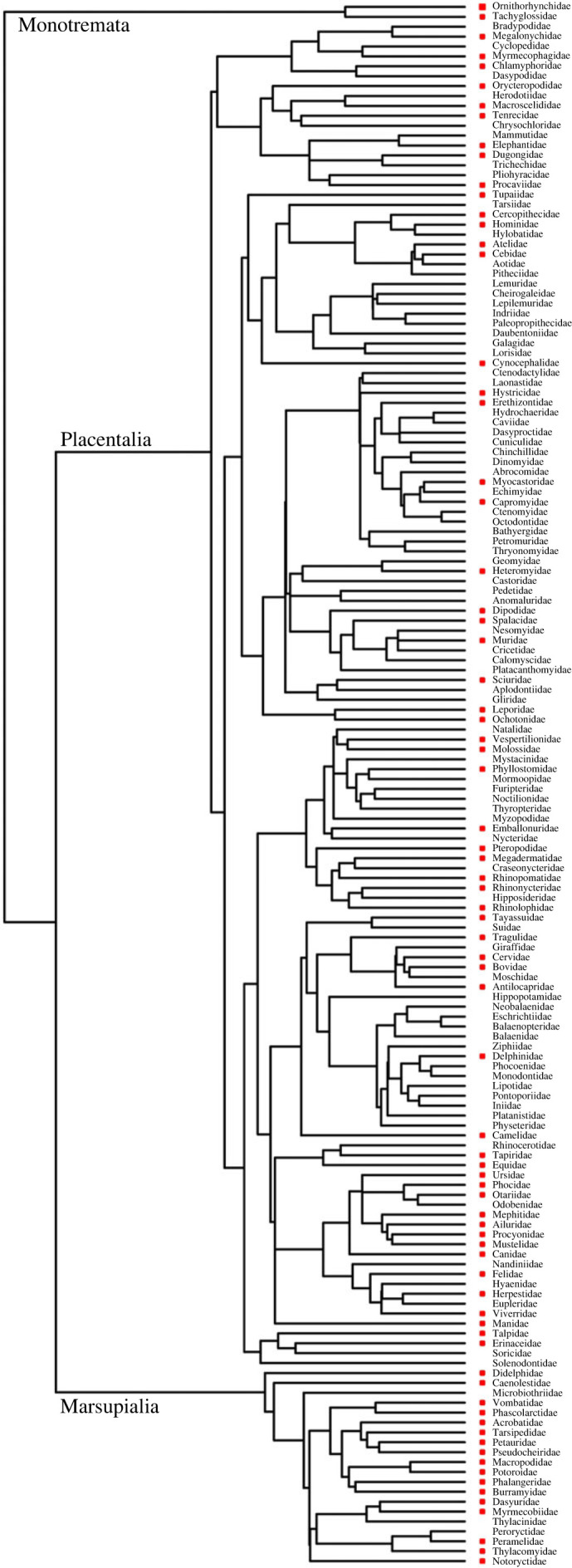
Phylogeny of mammalian families from Timetree.org [36]. A red square indicates those families for which we observed fluorescent species. The absence of a red squares indicates that fluorescence was not tested in this study.

Apparent fluorescence (our visual analysis of fluorescence was confirmed by spectroscopy for a subset of mammals in phase 1) was observed in all the mammal specimens investigated (figures 2 and 3, electronic supplementary material, tables S1 and S2). White fur was commonly fluorescent, along with lighter coloured fur (e.g. yellow, light brown; 107 out of 125 species). Naked skin inside the pinnae, around the eyes and mouth, inside marsupial pouches and on the feet was also fluorescent for many species (47 out of 125 species). Claws that contained pigment also appeared to fluoresce in some species (68 out of 125 species). The most fluorescent mammals were typically all white or pale yellow, such as the polar bear (*Ursus maritimus*), southern marsupial mole (*Notoryctes typhlops*) and an albino wallaby (*Notamacropus* sp.). Only one mammal had no external fluorescence, being the dwarf spinner dolphin (*Stenella longirostris roseiventris*) where only the teeth fluoresced. The frequency by which different body parts fluoresced was not uniform ($\chi_{7}^{2}=162$, *p* < 0.001), with white fur over-represented among fluorescent regions (1.75×), skin represented as expected (1.1×) and the other regions occurring at lower frequencies (0.23–0.89×). Our dataset had no clear phylogenetic bias (figure 3), indicating that fluorescence is common throughout the mammalian phylogeny.

#### 3.2.2. Analysis of the fluorescence presence

**Figure 4.**
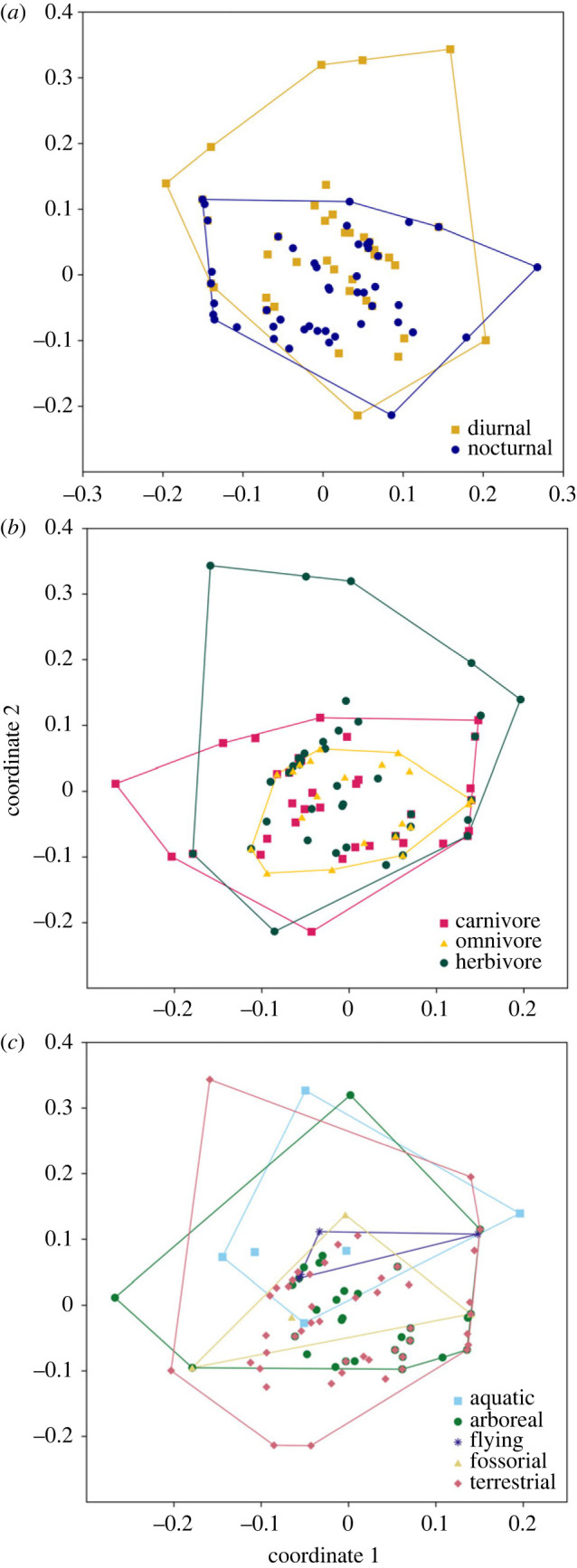
Principle coordinate analysis (PCO) of the presence and absence of fluorescence in 125 species of mammals.

The frequency of occurrence of different fluorescent colours was not uniform among mammals ($\chi_{5}^{2}=126$, *p* < 0.001). White (2.5×) and yellow (1.3×) colours occurred more frequently than expected while blue (0.5×), orange (0.3×) and pink (0.03×) occurred less frequency than expected (figure 2, electronic supplementary material, tables S1 and S2). Specimens with more regions of fluorescence were likely to be closer to the centre of the PCO plot, with least fluorescent specimens further from the centre. There was overlap between the plots for diurnal and nocturnal mammals (figure 4a; axis 1 = 23.57% of variance; axis 2 = 11.30% of variance) at the centre of the PCO plot, but there was less overlap at the extremities and only diurnal mammals were present at the top of the PCO (least fluorescent). Discriminant analysis of all 10 PCO scores for fluorescence achieved a highly significant separation between nocturnal (centroid −0.414) and diurnal (centroid 0.545) species with a single discriminate function incorporating 100% of the variance and with a canonical correlation score greater than or equal to 0.43 (*χ*_10_ = 24.4, *p* = 0.007).

For feeding guild (carnivore, omnivore, herbivores; figure 4*b*; axis 1 = 23.57% of variance; axis 2 = 11.30% of variance), both carnivores and omnivores occupy the centre of the PCO (more fluorescent), while herbivores were more widespread. Discriminate analysis of PCO scores for diet created two discriminate functions explaining 100% of the variance and with canonical correlation scores greater than or equal to 0.278; these functions could not significantly separate species of different diet (*χ*_9-20_ ≤ 21, *p* ≥ 0.396).

There was a lot of overlap between locomotor mode (figure 4*c*; axis 1 = 23.57% of variance; axis 2 = 11.30% of variance), but the centre (more fluorescent) of the PCO was mostly terrestrial, arboreal and fossorial mammals, while all locomotory modes occur in the upper half including aquatic and flying mammals. Discriminate analysis of PCO scores for locomotion types found three significant discriminate functions incorporating 97% of the variance and canonical correlation scores greater than or equal to 0.49 (*χ*_16-40_ ≥ 37.5, *p* ≤ 0.002). Aquatic species had the highest centroid for function one (3.32; less fluorescent), flying species function two (1.93; less fluorescent) and terrestrial species for function three (0.469; more fluorescent).

## 4. Discussion

To date, reports of fluorescence among mammals have been limited to a relatively small number of species [2,15,16,18,22–25,27]. Here, we were able to reproduce the results of these previous studies and observe apparent fluorescence in additional species; we report fluorescence for 125 mammal species, from half of all mammalian families and representing almost all clades in the mammalian phylogeny (figure 3). The only major mammalian clade missing from our dataset is the lemurs, a group that requires further investigation for the occurrence of luminescence; we predict, based on the prevalence of white fur, that this clade will also contain fluorescent species. While the amount and location of fluorescence varied between species, all exhibited some form of apparent fluorescence. Areas of fluorescence included white and light fur, quills, whiskers, claws, teeth and some naked skin. For some species (e.g. dwarf spinner dolphin) fluorescence was limited to the teeth, for which structural fluorescence is well known [37]. Likewise, white hair has previously been reported to fluoresce as a consequence of tryptophan or unpigmented keratin fibres [38–40]. It is therefore not surprising that pale-coloured quills, whiskers and claws also fluoresce, as they also consist of keratin. Porphyrin is associated with the red fluorescence in some mammals [18]. We observed red fluorescence in one quenda specimen (figure 2*h*), but not the other quenda examined. This corroborates the suggestion of Sobral and Souza-Gudinho [26] and Toussaint *et al*. [18] that red fluorescence was a result of a by-product of physiological processes unless it was faded out, but this requires further analysis.

Fluorescence of mammalian skin has not been widely reported, although Nummert *et al*. [25] observed florescence of dormouse skin. Mammalian skin is keratinized and so it is not surprising that we, Reinhold [41] and Nummert *et al*. [25] observed fluorescence on unpigmented skin of pinnae, face, marsupial and monotreme pouches, manus and pes. Nevertheless, we also observed fluorescence of pigmented regions of mammalian fur, suggesting that mammalian fluorescence is not just a consequence of the structural properties of unpigmented keratin, teeth and bone but the presence of fluorophores within the hair shaft as well as tryptophan residues in keratin [42].

The spectral curves we produced for glowing areas of a subsample of mammals representing monotremes, marsupials and placental mammals allow us to evaluate the apparent fluorescence and determine if the coloured ‘glow’ observed under UV light was probably fluorescence or the results of light scatter. Shifting the excitation wavelength did not produce a corresponding shift in the peak of these reflectance curves, indicating that the observed phenomena is indeed fluorescence and not just scatter [32]. The concurrent observed drop in intensity is expected, as the excitation is no longer at the optimal wavelength, depending on the fluorophore targeted. Consequently, we directly confirm previous observations of the presence of fluorescence for the platypus [24] and, based on the taxonomic and ecological diversity of our mammalian subset, suggest that other reports of fluorescent mammals are likely to be a consequence of actual fluorescence and not light scatter.

Previous studies have demonstrated that the fluorescence observed for some preserved museum specimens also occurred in live animals [2,22,23,25], but fluorescence was not quantified in these studies. Our spectral curves, especially for the platypus, where we explored two forms of preservation and a frozen specimen, provide quantitative evidence that both preserved and non-preserved specimens fluoresce, and that for all three light scatter is not the cause of the coloured ‘glow’ observed under UV light. However, borax itself also fluoresces, suggesting that preservation may play a part in the intensity of the fluorescence observed for some specimens. Indeed, the fluorescent property of borax is one reason it is widely used as a cleaning agent to brighten white clothing and other materials. Interestingly, Pine *et al*. [16], Olson *et al*. [23] and Nummert *et al*. [25] reported a decrease in fluorescence intensity for preserved compared with live dormice while Kohler *et al*. [22] reported a similar degree of fluorescence for live and preserved flying squirrels, but they were tested in different conditions. Storage conditions over the life of preserved specimens may impact the intensity of fluorescence observed; newer specimens protected from light may retain fluorescent characteristics if fluorescent molecules are photodegradable [16,18].

There is some debate as to whether fluorescence in mammals, and indeed other animals, has an actual biological function, or if it is simply a consequence of the nature of their surface chemistry (e.g. [1,43,44]). Marshall and Johnsen [1] suggested that assigning a biological function to observations of fluorescence requires five criteria: that the absorption spectra of fluorescent pigments absorb available light wavelengths, under what natural lighting condition it is, emitted wavelengths contrast against typical backgrounds, fluorescent areas of animals are visible, and intended observers have an appropriate spectral sensitivity. They concluded that it can be rarely demonstrated that all these criteria are met for most examples of fluorescence, and so there was little evidence that most of the fluorescence described has a biological function. For most fluorescent mammals there is insufficient information to evaluate if they conform to these five criteria, so we can only hypothesize about potential roles, if any, of mammalian fluorescence with the aim to stimulate further investigation into the potential functions of what we now understand is a widespread phenomenon.

The presence of white or light fur, which we found was more likely to fluoresce, was a major driver for the extent of fluorescence in our broad mammalian dataset. The evolution of coloration in mammals is complex, with many drivers of colour and pattern including camouflage, signalling and physiology [45]. It is likely that much of the fluorescence we recorded is the result of tryptophan metabolite fluorophores that are otherwise masked by melanin in darker fur [42]. The interaction between day/night use, feeding guild and locomotion is probably important for determining the extent of white fur in mammals, and fluorescence is likely to be simply a consequence of unpigmented fur for many species.

Previous studies have suggested a link between mammalian fluorescence and nocturnality [2,22–24]. Our results support this suggestion with the extent of fluorescence discriminating between nocturnal and diurnal mammals. As very few of the mammals in our dataset had pink-, orange- or red-coloured fluorescence, it is unlikely that a predominance of glandular secretions, such as might be associated with a greater reliance on olfactory communication, explains a predominance of fluorescing nocturnal species. However, degradation of pigments which produce these reddish colours over time for our museum specimen dataset (e.g. [16,18]) may account for the significant under-representation of these fluorescent colours in our dataset. Despite fluorescence being more widespread for nocturnal species, fluorescence was not restricted to these species and we identified some 52 diurnal mammals that also fluoresce, albeit typically in fewer body regions.

For nocturnal mammals, white or light-coloured areas may allow for intra- and inter-specific communication [46,47]; fluorescence of these areas increases brightness, enhancing visibility and therefore presumably value as visual signals especially as the moon becomes fuller, reflecting more sunlight. During twilight, there is a shift to shorter wavelength ambient light due to attenuation by the atmosphere of medium wavelengths from a low solar angle [48], so during these low-light periods, areas that emit light at longer wavelength (i.e. that fluoresce) are likely to be more visible. Penteriani and Delgado [47] suggest that visual signalling among nocturnal and crepuscular mammals is more important than previously recognized and our observations of widespread fluorescence, particularly among nocturnal species, supports this conclusion.

In terms of locomotion, we found the broadest occurrence of fluorescence within terrestrial, arboreal and fossorial mammals, with flying and aquatic mammals less likely to fluoresce. Although the platypus had been reported as fluorescing [24], our results indicate that the intensity of fluorescence in a fresh specimen was lower than for preserved specimens, suggesting a minor, if any, functional role. Platypus hunt for aquatic prey underwater with their eyes closed [33], so their ventral fluorescence, if it has any functional purpose, is unlikely to be a visual cue, as it is only likely to be visible when they are in the water in a three-dimensional environment. It may provide some benefit in brightening the ventral surface to provide counter shading in water, as is common for many aquatic or marine species [49]. It is also unlikely that fluorescence plays a functional role for fossorial mammals. The southern marsupial mole (*Notoryctes typhlops*) is one of the most fluorescent mammals, covered in yellow-white fur, but it is also blind [33]. Its fluorescence is probably a result of reduced pigmentation that occurs in many fossorial and subterranean species [50] or of increased structural keratin to protect against abrasive soil particles, as hypothesized for the golden mole [51]. Fluorescence is unlikely to be important for some flying mammals such as microbats that use echolocation to navigate and locate prey.

## 5. Conclusion

In this study, we have demonstrated widespread fluorescence among mammals, by confirming the phenomenon using spectral analysis and subsequently examining observable fluorescence throughout the phylogeny of mammals. We have identified many candidate species for further examination by spectral analysis, and present evidence that preservation methods could impact these results by increasing or decreasing the intensity of the fluorescence. We would suggest that further studies should focus on non-preserved animals, e.g. live or freshly dead, as these would not be impacted by potential degradation of fluorescent materials or by preservation chemicals. Species of interest would include those with highly patterned pelts, which may be important for visual signalling or camouflage, and those with highly specialized life-histories.

## Data Availability

All data are provided in the supplementary figures and tables, and the raw data are provided as Excel files [52].
